# Effects of chitosan-containing silver nanoparticles or chlorhexidine as the final irrigant on the bond strength of resin-based root canal sealers

**DOI:** 10.34172/joddd.2022.020

**Published:** 2022-10-15

**Authors:** Berkan Celikten, Gulin Amasya, Aysenur Oncu, Mehrdad Koohnavard, Feridun Saklar

**Affiliations:** ^1^Faculty of Dentistry, Department of Endodontics, Ankara University, Ankara, Turkey; ^2^Faculty of Pharmacy, Department of Pharmaceutical Technology, Ankara University, Ankara, Turkey

**Keywords:** Chitosan, Chitosan-silver nanoparticle, Chlorhexidine gluconate, Irrigation solutions

## Abstract

**Background.** This study evaluated the combined effects of silver nanoparticles (AgNPs) and chitosan on the dentin bond strength of resin-based root canal sealers using the push-out test and scanning electron microscopy (SEM).

**Methods.** This in vitro study was conducted on 72 extracted mandibular premolar teeth. All the teeth were decoronated perpendicular to the long axis to leave a 13-mm root length. The root canals were prepared, and the samples were randomly divided into seven experimental groups and one control group based on final irrigation solutions. All the final irrigation procedures were performed for one minute. The root canals were dried using paper points and filled with a resin-based sealer and gutta-percha points using a lateral condensation technique. Sections measuring 2 mm in thickness were taken from the apical, middle, and coronal thirds of each root using a cutting machine. The push-out test was performed using a universal testing machine.

**Results.** The solution of AgNPs combined with 0.4% chitosan showed higher bond strength in the coronal region than a combination with 0.2% chitosan. Samples treated with 0.4% chitosan solution exhibited a higher bond strength than the 0.2% chitosan group. There were no significant differences between chlorhexidine (CHX) solution alone and in combination with 0.2% or 0.4% chitosan solution.

**Conclusion.** The combination of chitosan and AgNPs was as effective as CHX in improving the bond strength of resin-based sealers.

## Introduction

 Effective endodontic treatment requires close adaptation of the root canal filling material to dentinal walls. Removal of the smear layer and adhesion of the root canal sealer to dentinal tubules enhances the obturation quality. The smear layer contains debris generated during root canal preparation, microorganisms, and metabolic products. This layer can prevent the penetration of intracanal medicaments and disinfectants into the dentinal tubules and compromise the adaptation of endodontic materials to dentinal walls.^1^ Various irrigants are used to prevent this, and the final irrigant is particularly important for both disinfection and removal of the smear layer.^2,3^

 Chlorhexidine (CHX) is a biocompatible disinfectant with a broad spectrum of antimicrobial activity. This widely used chemical agent is generally preferred to treat recurrent periapical infections.^4^ CHX can also improve root canal sealing by reducing the activity of collagen-disruptive matrix metalloproteinases in radicular dentin.^5^

 Chitosan, a natural polysaccharide derived from crab and shrimp shells, is used as a chelating agent.^6^ Chitosan is a remarkable endodontic irrigant due to its bioactivity, selective permeability, and antimicrobial effects.^7^ Previous studies have shown that chitosan increases the bond strength between the root canal sealer and dentinal tubules.^6,8^

 Silver nanoparticles (AgNPs) are a new material with unique surface properties and high antimicrobial efficacy.^9^ AgNPs have become popular because they release silver ions, which cause bacterial cell deterioration. In addition, it has been reported that pretreatment of dentin with AgNPs increases the bond strength in adhesive systems.^10^ This agent might be a useful final irrigant due to enhanced dentin bonding and disinfection.

 The push-out test is a reliable method to determine the bond strength of materials. Scanning electron microscopy (SEM) allows detailed examination and visualization of the root canal filling and dentin interface. This study aimed to evaluate the combined effect of AgNPs and chitosan on the bond strength of resin-based sealers with dentin using the push-out test and SEM.

## Methods

###  Sample selection and preparation

 This in vitro study was conducted using 72 extracted mandibular premolar teeth. Intact, single-rooted, and closed-apex samples were selected from teeth with similar corono-apical dimensions (20–23 mm) that were extracted for reasons unrelated to the study. Residual debris and soft tissues on the teeth were removed using periodontal curettes, and the teeth were kept in distilled water containing 0.1% thymol crystals at room temperature. This study was approved by the Ethics Committee of Ankara University.

 All the teeth were decoronated perpendicular to the long axis (13 mm of root length) using a high-speed, water-cooled handpiece. The root canals were prepared up to #F3 using the ProTaper rotary file system (Dentsply Sirona, York, PA, USA). Instrumentation was performed by an operator using an Endomotor (X-Smart; Dentsply Sirona). The root canals were irrigated with 5.25% sodium hypochlorite (NaOCl) solution during the preparation procedure for lubrication and removal of debris. The samples were randomly divided into seven experimental groups and one control group based on the final irrigant.

###  Preparation of chitosan solution and chitosan- AgNP dispersion

 Chitosan solution (0.2% and 0.4%, w/v) was prepared by mixing medium-weight chitosan powder in 1% v/v acetic acid aqueous solution and filtered through a membrane (0.45 μm) to eliminate insoluble fractions.

 The chitosan-AgNP was synthesized using the chemical reduction method. Stock silver nitrate (AgNO_3_) aqueous solution and stock sodium borohydride (NaBH_4_) solution were prepared.^11^ For 25 mL of chitosan-AgNP dispersion, 1 mL of 0.01-M AgNO_3_ aqueous solution was mixed with 23.5 mL of 0.2% or 0.4% chitosan solution and incubated at 50°C for 30 minutes with shaking. Then, 0.5 mL of freshly prepared NaBH_4_ solution was added and stirred for 90 minutes to obtain a clear yellow-to-brown color.

**Figure 1:**
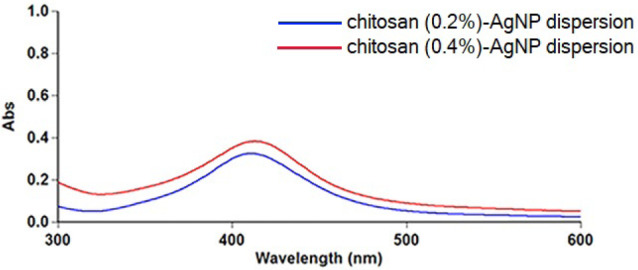
UV absorbance of chitosan-silver nanoparticles.

 The synthesized chitosan-AgNP was physicochemically characterized using a UV-visible spectrophotometer (Cary 60 UV-Vis; Agilent Technologies, Inc., Santa Clara, CA, USA) and transmission electron microscopy (TEM; G2 S Twin; FEI, Hillsboro, OR, USA) (Figure 1).

###  Treatment of samples and push-out test protocol

 The samples were treated with 2% CHX solution (group 1), 0.2% chitosan solution (group 2), 0.4% chitosan solution (group 3), 0.2% chitosan-AgNP dispersion (group 4), 0.4% chitosan-AgNP dispersion (group5), 0.2% chitosan solution containing 2% CHX (group 6), or 0.4% chitosan solution containing 2% CHX (group 7). Distilled water was used for the control group. All the final irrigations were performed for one minute. The root canals were dried using paper points. Subsequently, the teeth were filled with a resin-based sealer (AH Plus; Dentsply Sirona) and gutta-percha points using the lateral condensation technique. One sample from each group was selected for SEM analysis.

 Then, 2-mm sections were taken from the apical, middle, and coronal thirds of each root using a cutting machine. The push-out test was performed using a universal testing machine (Lloyd Instruments Ltd., Bognor Regis, UK). The dentin discs with root canal fillings were set in the mechanical test machine with a 1-kN load cell. Progressive compression testing was performed with force applied in the corono-apical direction at a crosshead speed of 1 mm/min, between the device tip contact and the filling material displacement.

###  Stereomicroscopic evaluation

**Figure 2:**
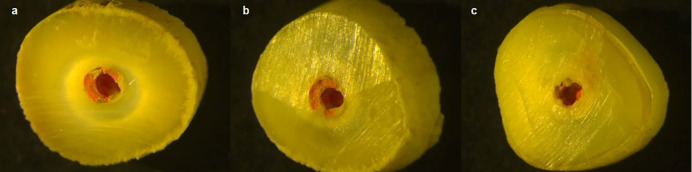
Stereo-microscope images of samples (a: A sample with mixed failure in the coronal section; b: A sample with mixed failure in the middle section; c: A sample with adhesive failure in the apical section).

 All the specimens were observed under a stereomicroscope (Leica Microsystems, Wetzlar, Germany) for failure mode diagnosis. The failure modes were classified based on the relation of the gutta-percha and root canal sealer with the dentinal wall. Adhesive failure was defined as complete removal of the root canal filling material; cohesive failure was defined as root canal filling material remaining on dentinal walls; and mixed failure was defined as partial removal of root canal filling material from dentinal walls. Adhesive and mixed failures were the most common types. Failures in the coronal, middle, and apical thirds are shown in Figure 2 as an example.

###  SEM protocol

**Figure 3:**
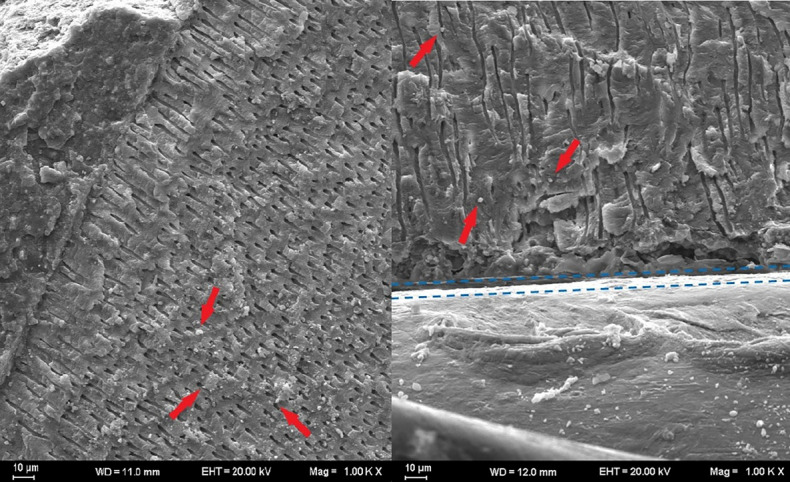
SEM images of the dentin surface (red arrows: root canal sealer particles; blue dashed lines: border between the root canal filling and the dentin).

 Samples were examined with a scanning electron microscope to visualize the depth of penetration of the root canal sealer. One sample from each group was selected and prepared for SEM examination. The specimens were marked using a diamond disk and divided longitudinally into two sections; both sections were used for SEM analysis. The prepared samples were sputter-coated and placed into the vacuum chamber of the scanning electron microscope (Carl Zeiss NTS GmbH, Oberkochen, Germany). Sealer penetration and adaptation were examined from the coronal to apical ends at ×1000 magnification, and microphotographs were taken (Figure 3).

###  Statistical analyses

 Statistical analyses were performed using SPSS 20.0.1 (IBM Corp., Armonk, NY, USA). The normality of the data distribution was examined using the Kolmogorov–Smirnov test, while homogeneity was assessed using Levene’s test. Comparison of group means was performed using one-way ANOVA. Post-hoc analyses were performed using Tukey and Bonferroni tests. *P* < 0.05 indicated statistical significance.

## Results

 The particle size of chitosan-AgNP was evaluated by UV–visible spectrophotometer at approximately 300–600 nm. AgNP dispersions with 0.2% and 0.4% chitosan showed absorption peaks at 410 and 413 nm, respectively. AgNPs showed strong surface plasmon resonance (SPR) absorption peaks of about 390–470 nm, which depended on size, shape, and distribution.^12,13^ According to the results of UV-visible spectrophotometry, the particle size of chitosan-AgNP ranged from 15 to 20 nm due to the surface plasmon energy. TEM analyses were carried out to confirm these results, and the morphologies of the chitosan-AgNPs were observed at an acceleration voltage of 200 kV at room temperature.

**Figure 4:**
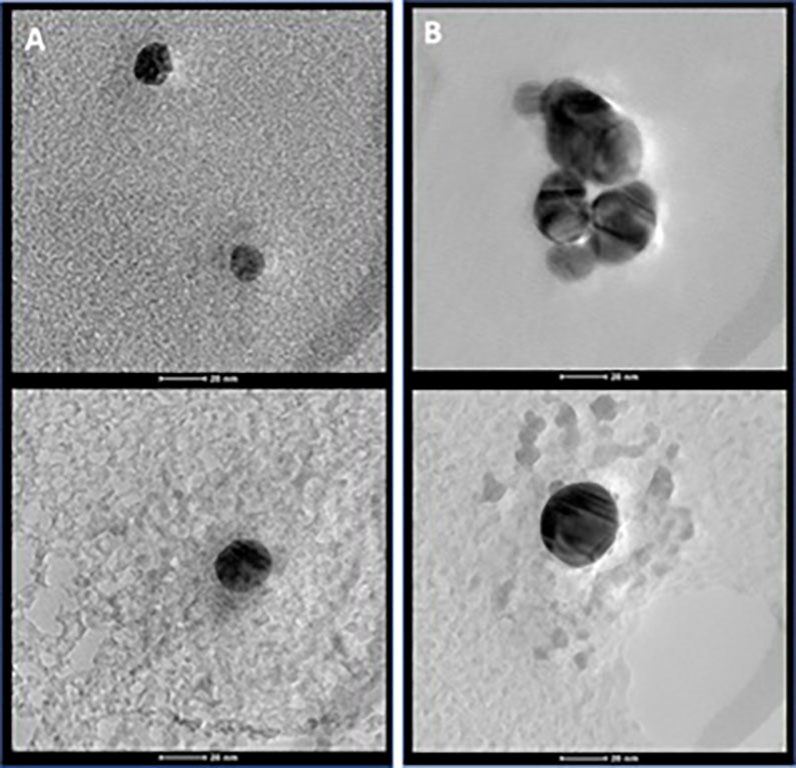
Transmission Electron Microscopy images of chitosan - silver nanoparticles (a: prepared with 0.2% chitosan solution; b: prepared with 0.4% chitosan solution).

 TEM images of both chitosan-AgNPs demonstrated spherical and smooth shapes with no aggregation (Figure 4). Moreover, the TEM micrographs revealed particles of around 20 nm in size, consistent with the UV-visible spectrophotometry analysis.

 All the specimens had measurable adhesion to root dentin, and no premature failures occurred. The mean bond strength values were expressed in megapascals (Table 1). Groups 1, 3, 5, 6, and 7 showed significantly higher values in the coronal region than the other groups. Group 3 exhibited higher bond strengths compared to group 2. Group 5 showed higher bond strength in the coronal region than group 4. There were no significant differences between CHX solution alone and CHX in combination with 0.2% or 0.4% chitosan solution. The highest bond strengths were observed in the apical sections. Middle sections showed higher bond strength than coronal sections in all the groups (*P* < 0.05). However, there were no significant differences between the experimental groups (*P* > 0.05).

**Table 1:** The mean bond strength values in megapascal

**Final irrigation solution **	**Groups **	** Coronal**	** Middle**	** Apical**
2% Chlorhexidine gluconate	Group 1	3.20^A^	6.03^B^	11.79^C^
0.2% chitosan solution	Group 2	1.88^a^	4.84^B^	10.48^C^
0.4% chitosan solution	Group 3	3.23^A^	6.91^B^	11.9^C^
Chitosan (0.2%) - silver nanoparticle dispersion	Group 4	1.74^a^	5.65^B^	10.40^C^
Chitosan (0.4%) - silver nanoparticle dispersion	Group 5	3.31^A^	6.30^B^	14.51^C^
0.2% chitosan solution containing 2% chlorhexidine gluconate	Group 6	3.35 ^A^	6.25^B^	14.04^C^
0.4% chitosan solution containing 2% chlorhexidine gluconate	Group 7	3.45 ^A^	7.16^B^	14.86^C^
Distilled water	Group 8	1.31^a^	2.95^b^	6.2^c^

*Note:* Same superscript letters indicate no statistical difference. Different superscript letters indicate statistical significance. There is statistical significance between small and capital superscript letters.

## Discussion

 The effects of final irrigants on the bond strength of resin-based root canal sealers were investigated in the present study. Chitosan is considered an alternative to ethylenediaminetetraacetic acid (EDTA) because of its antibacterial and physiochemical properties, such as high chelating ability in acidic conditions, biocompatibility, and biodegradability.^14^ Antunes et al^15^ found that 0.2% chitosan and 15% EDTA were equally effective. Chitosan, a natural substance, is more beneficial for root canal treatments because EDTA is erosive and destructive.^16^ Therefore, we used two different concentrations of chitosan. In coronal sections, 0.4% chitosan solution showed better bond strength than 0.2% chitosan solution. However, the concentration did not affect the bond strength in the middle or apical section. A previous study demonstrated that teeth pretreated with 0.2% chitosan had 17% higher bond strength with bioceramic sealers compared to teeth pretreated with EDTA.^17^

 CHX is still a commonly used endodontic irrigation solution for persistent infections. Previous studies demonstrated that CHX improves the bond strength of resin-based cements and composite resins to dentin.^18,19^ In the present study, 2% CHX and 0.4% chitosan showed similar results in all the sections. There was no significant difference in the bond strength of CHX combinations with chitosan between two different concentrations. Dinesh et al^20^ reported that 2% CHX improved the bond strength of root canal fillings using AH Plus sealer. Our findings showed that all the CHX-containing groups had higher bond strengths than the control group. Irrigation with 2% CHX significantly reduced the contact angle between the surface and resin-based sealer, increasing its dentinal adhesion regardless of the presence of the smear layer.^21,22^

 AgNPs are used in dentistry because of their unique antimicrobial properties, which result from silver ion release.^10,23^ A combination of chitosan and AgNPs may further increase this antimicrobial activity.^24^

 In this study, the disinfectant properties of AgNPs and the chelating properties of chitosan were combined to produce a “two-in-one” final irrigant. Samples pretreated with 0.4% chitosan-AgNP solution showed bond strengths similar to the CHX groups. No studies have measured the bond strength of root canal sealers when AgNPs were used as final irrigants. However, few researchers have studied the bonding of luting cements after treatment of post cavities with AgNP solution. Shafiei et al^25^ and Jowkar et al^10^ demonstrated that pretreatment of intraradicular dentin with AgNPs improved the post system adhesion. Another study investigated the fracture resistance of nanoparticles in endodontically treated teeth. Silver, zinc oxide, and titanium dioxide nanoparticles showed greater fracture resistance than conventional solutions.^26^ Suzuki et al^27^ reported that AgNP irrigation improved the mechanical properties of glass fiber after cementation. Our findings are consistent with these studies, which established the positive effects of AgNP irrigation.

## Conclusion

 This study introduced a new final irrigation solution with 0.4% chitosan containing AgNPs to improve the bond strength of resin-based root canal sealers. A final irrigation protocol using CHX, chitosan, and their combinations improved the adaptation of root canal filling material to the dentinal walls.

## Authors’ Contribution

 AO and MK were responsible for the experiment design, performed the experiments to fulfill the requirements for a degree, and wrote the manuscript. GA was responsible for the experiment design and contributed to the discussion. Data entry and statistical analyses were carried out by MK and AO. All the images were evaluated and measured by FS. BC conceived the idea, hypothesis, and experiment design. All authors have read and approved the final manuscript.

## Funding

 This study was conducted with the resources of the authors.

## Ethics Approval

 This study was approved by the Ethics Committee of Ankara University Faculty of Dentistry.

## Competing Interests

 The authors declare no conflicts of interest related to the publication of this work.
